# Navigating the Chemical Space of Multitarget-Directed Ligands: From Hybrids to Fragments in Alzheimer’s Disease

**DOI:** 10.3390/molecules21040466

**Published:** 2016-04-08

**Authors:** Federica Prati, Andrea Cavalli, Maria Laura Bolognesi

**Affiliations:** 1 Department of Pharmacy and Biotechnology, Alma Mater Studiorum—University of Bologna, Via Belmeloro 6, I-40126 Bologna, Italy; federica.prati3@studio.unibo.it; 2 Sir James Black Centre, College of Life Sciences, University of Dundee, Dundee DD1 5EH, UK; 3 Department of Drug Discovery and Development, Istituto Italiano di Tecnologia, Via Morego 30, 16163 Genova, Italy

**Keywords:** multitarget drug discovery, galantamine, memantine, donepezil, clioquinol, BACE-1 inhibitors, GSK-3β inhibitors

## Abstract

Multitarget drug discovery is one of the hottest topics and most active fields in the search for new molecules against Alzheimer’s disease (AD). Over the last 20 years, many promising multitarget-directed ligands (MTDLs) have been identified and developed at a pre-clinical level. However, how to design them in a rational way remains the most fundamental challenge of medicinal chemists. This is related to the foundational question of achieving an optimized activity towards multiple targets of interest, while preserving drug-like properties. In this respect, large hybrid molecules and small fragments are poles apart. In this review article, our aim is to appraise what we have accomplished in the development of both hybrid- and fragment-like molecules directed to diverse AD targets (*i.e.*, acetylcholinesterase, NMDA receptors, metal chelation, BACE-1 and GSK-3β). In addition, we attempt to highlight what are the persistent needs that deserve to be improved and cared for, with the ultimate goal of moving an MTDL to AD clinical studies.

## 1. Introduction

The recent appreciation of the network medicine concepts has had significant implications for drug discovery, leading to the new era of multitarget drugs [1,2]. Network medicine is the emerging science that takes into account the network effects of biological and medical occurrences [3]. From the network medicine perspective, diseases are the results of the systemic breakdown of physiological networks, due to the suppression or activation of certain stages and a consequent imbalance of input-output [4]. Thus, the goal of therapy is to restore the perturbed disease networks by simultaneously targeting key components/checkpoints by drugs. However, diseased networks are difficult to repair through intervention at a single node (protein target/signaling pathway), because robust and redundant mechanisms are typical of complex biological network systems. It thus follows that the modulation of several drug targets through a well-concerted polypharmacological approach is essential to achieve the desired therapeutic effect [5].

We were contributors to an early debate on the potential of this approach in the field of neurodegenerative drug discovery and, particularly, in Alzheimer’s disease (AD) [6]. Our conviction was based on the fact that “drugs hitting a single target may be inadequate for the treatment of diseases like neurodegenerative syndromes, which involve multiple pathogenic factors” [6]. The discovery of memoquin, one of the first AD drug candidates rationally designed using a multitarget approach, amply confirmed the initial assumption [7].

Over the years, this founding principle, which is applicable to many other currently incurable diseases sharing a multifactorial pathology, received strong support by the considerations that are instead peculiar to AD treatment. A critical issue is that elderly AD patients are susceptible to a wide range of concomitant co-morbidities (e.g., hypertension, vascular diseases or diabetes). This means that they are subject to major polypharmacy with a greater risk of drug-drug interaction and toxicity. There is therefore a need for therapeutic tools to be tailored to their specific conditions [8]. A so-called multitarget-directed ligand (MTDL) is one such tool, as it abolishes the risk of drug-drug interaction. Furthermore, simplifying the therapeutic regimen with a single multitarget agent could add significant value for dementia patients and their caregivers who have inherent difficulty with compliance and therapy adherence [8].

**Figure 1:**
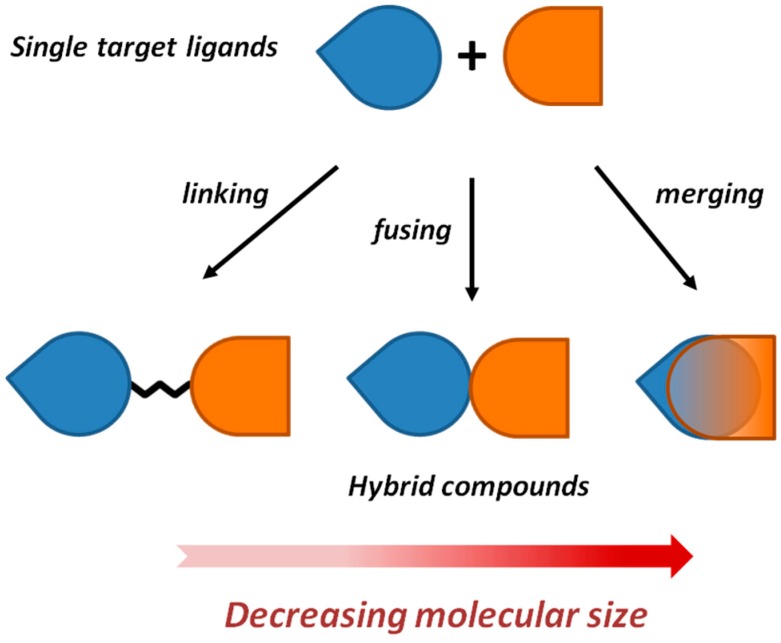
*Framework combination* of two single-target ligands into a new dual-targeted chemical entity through *linking*, *fusing* and *merging* approaches.

Despite solid rationale and suggestive early-published studies, it was soon evident to medicinal chemists that the design of multitarget drugs was not an easy task [9]. To this end, pioneers Morphy and Rankovic delineated two possible broadly different strategies: a random screening approach and a *framework combination* approach [10]. In the first case, compound classes that are already known to be active against one of the targets of interest are screened against the other target in an unbiased fashion. Conversely, *framework combination* is a knowledge-based approach, which aims to combine two molecular frameworks into a new single dual-targeted chemical entity. The authors coined the terms *linking*, *fusing* or *merging* to differentiate to what extent the two starting frameworks are integrated [11,12] (Figure 1). At one end of the spectrum, there are conjugated ligands, which contain distinct frameworks connected by a linker. Ligands designed in this way are more likely to have high molecular weight (MW) and less likely to have oral drug-like properties. At the other end of the spectrum, there are ligands in which molecular frameworks overlap or are highly integrated (*i.e.*, *fused* or *merged*). Such compounds are likely to have lower MW and potentially better drug-like properties.

All this clearly highlights that the rational design of multitarget drugs has to deal with the crucial issue of achieving an optimized activity towards the targets of interest, while maintaining drug-like properties. An analysis performed by the same authors already in 2006 revealed that the reported MTDLs were typically larger and more complex than conventional single-targeted preclinical ligands [12]. One explanation for this was the popularity of the *framework combination* approach as a commonly-followed design strategy. As the selective starting ligands were already drug-like and the extent of the framework overlap that could be reached was in many cases low, this process resulted in large property increases that often compromised oral bioavailability [12].

Against this backdrop, over the years, fragment-based drug discovery (FBDD) strategies have received increasing consensus as optimal starting points for multitarget drug design [13,14]. In this respect, in 2001, Hann and co-workers calculated the probability of interaction between proteins and ligands of diverse molecular complexity. They reached the conclusion that smaller molecules were better starting points for drug discovery. This is because the lower the complexity of a molecule, the higher its chance of recognizing biological targets [13]. Several years later, by analysing the Pfizer corporate screening data, Hopkins discovered a clear inverse correlation between mean MWs and promiscuity. This was explained as evidence that smaller molecules, having less negative interacting features, are more likely to establish interactions with multiple targets [9]. In other words, less complex molecules are more prone to bind to multiple proteins due to the lower probability of a mismatch between ligand and the proteins of interest. Along the same lines, Morphy’s analysis of Organon’s SCOPE database revealed a clear correlation between size and selectivity [15], supporting the hypothesis that the inherent simplicity of small molecules favours non-selective binding events [16]. Moreover, there is ample experimental support for a higher hit rate for fragments in multitarget screening with respect to the larger compounds typically screened in high throughput screening (HTS). In this respect, FBDD might also provide more drug-like structures than the large and lipophilic molecules identified through HTS, thus simplifying physicochemical issues related to the hit- and lead-optimization process [16].

Following the identification of a base scaffold able to bind to multiple targets, subsequent growing could provide a ligand with higher affinity at each target, whereas maintaining high ligand efficiency (LE). While it is highly conceivable to identify multitarget fragment hits, the challenge with the FBDD will be to improve and balance activity at each target. As for single-target FBDD, structural information will be critical to drive the optimization process. However, if the targets are too different, it may not be possible to grow the fragment in a way that simultaneously improves affinity at each target [17]. Nevertheless, the extent to which affinity must be improved may be lower for an MTDL than for a single-targeted molecule, if strong pharmacodynamic synergy between the targets exists [16].

Regarding practical application of FBDD in multitarget drug discovery, in 2009, an efficient fragment-screening approach to peroxisome proliferator-activated receptor (PPAR) MTDLs was reported [17]. Furthermore, a fragment screening campaign directed at two very different enzyme targets, such as the c-Jun *N*-terminal kinase 3 (JNK3) and β-glucosidase, has been also successfully carried out [17]. In aggregate, these findings suggest a potentially wide applicability of FBDD in multitarget drug discovery.

In some ways, the FBDD approach represents the antithesis of *framework combination*, in that they cover different regions of the multitarget chemical space. Certainly, if we consider MW as the descriptor on one axis of this chemical space, the different position of small fragments (<300 Da) and large hybrid compounds (>500 Da) is easily apparent.

In pursuit of novel MTDL candidates against AD, we have adopted both *framework combination* and FBDD strategies. The two were carried out in parallel obtaining interesting, still preliminary, results. Indeed, we believe that they are not mutually exclusive, but are rather complementary when searching for MTDLs, each with its own pros and cons. Accordingly, the rest of this review is divided into two parts dealing with case studies provided by our research and inspired by the two approaches.

## 2. Framework Combination Strategies Applied to the Discovery of Hybrid Anti-AD Lead Candidates

### 2.1. Development of Memantine-Galantamine Hybrids via a Linking Strategy

As anticipated, the most common strategy to achieve a multitarget profile against AD is to connect distinct pharmacophores in the same hybrid compound. Theoretically, each pharmacophore of this new hybrid entity should still bind with its specific site(s) on the target, thus producing specific pharmacological responses that might synergistically modulate the pathologic cascade underlying neurodegeneration. Looking at the specific reported examples, most hybrids inhibit cholinesterases (ChEs) and concomitantly interact with one further pharmacological target implicated in AD pathogenesis, such as the H3, M1, NMDA or 5-HT4 receptors. Enzymes like β-secretase-1 (BACE-1), monoamine oxidase (MAO) and the serotonin transporter have been targeted. In addition to specific protein targets, developed hybrid molecules can also incorporate antioxidant or anti-amyloid fragments [18]. Several review articles describing the status and advances of this class of MTDLs have been recently published, to whom the reader is directed for a more comprehensive discussion [19,20,21,22,23,24].

**Figure 2:**
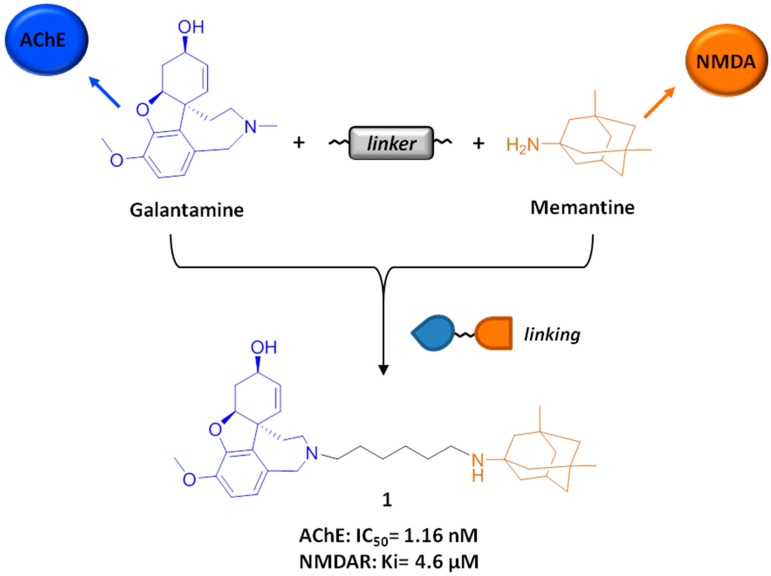
Design strategy (*linking* approach) and multitarget activity of **1**.

By following a *conjugation* approach, in 2012, some of us developed a new series of hybrid compounds that integrated the pharmacological activities of two drugs marketed for AD, which are the acetylcholinesterase inhibitor (AChEI) galantamine and the *N*-methyl-d-aspartate receptor (NMDAR) antagonist memantine [25] (Figure 2). The design aim was to combine in the same molecule the neuroprotective effect of NMDAR antagonism with the symptomatic relief provided by cholinergic neurotransmission through acetylcholinesterase (AChE) inhibition. In fact, drug combination targeting both the cholinergic and glutamatergic systems is the current standard of care for AD patients [26]. The underpinning rationale for such a combination is that the NMDAR antagonist could contrast neurodegeneration, while the AChEI could restore memory and cognition by stimulating still alive neurons. Additionally, it is well documented that the glutamatergic and cholinergic neuronal systems influence each other and that their joint dysfunction is crucial in AD pathology [27]. On this basis, galantamine and memantine, working together on the same excitotoxic cascade, could provide a synergistic neuroprotective effect.

Therefore, novel dual-target compounds were developed by connecting the two drugs through variable-length polymethylene spacers and heteroatom linkers [25] (Figure 2).

Despite previous studies having already reported on dual AChE/NMDAR compounds [28,29], this was the first time that two marketed drugs were rationally combined in a single new chemical entity.

The new hybrid compounds were initially conceived of to simultaneously bind both the catalytic and peripheral anionic sites (CAS and PAS) of the AChE gorge, following a dual-binding approach, and to interact with NMDAR. Interestingly, **1** (memagal in Figure 2) carrying a hexamethylene spacer resulted in one of the most promising compounds of the series. In agreement with molecular modelling studies, six methylenes constituted the optimal distance to allow **1** to efficiently contact the AChE CAS and PAS, resulting in a nanomolar AChE inhibitor (IC_50_ = 1.16 nM) [25].

As for the NMDA activity, **1** showed micromolar affinity to NMDAR (Ki = 4.6 µM) in the assay based on the displacement of the [^3^H] radiolabelled NMDA antagonist MK-801. More importantly, **1** resulted in binding to the NR2B subunit of NMDAR (Ki = 4.6 µM in the [^3^H]-ifenprodil binding assay), which is considered the major contributor of glutamate excitotoxicity [30,31,32].

Outstandingly, **1** also displayed prominent neuroprotective cellular activity, inhibiting NMDA-induced neurotoxicity in neuroblastoma cells with an IC_50_ value of 0.28 nM [25]. Considering that the observed neuroprotective profile of **1** could not be fully ascribed to its micromolar NR2B binding potency, it was inferred that the galantamine moiety of **1** also contributed to neuroprotection [33]. Based on previous evidence [34,35], this effect is likely to be associated with the activity of galantamine on nicotinic receptors, rather than on NMDAR. Indeed, it is quite conceivable that the interplay of NMDA and nicotinic receptor modulation, mediated by memantine and galantamine, respectively, delivered the remarkable neuroprotective potency of **1**.

Overall, this study describes a rationally-designed molecule with a dual AChE/NR2B profile and illustrates a successful and interesting strategy for developing conceptually new MTDLs, as possibly more effective and safer drug treatments over the current drug combinations commonly used in clinical practice. On this basis, *in vivo* studies to investigate the multitarget profile of **1** have been undertaken and will be reported in a separate paper.

### 2.2. Development of Clioquinol-Donepezil Hybrids via a Fusing Strategy

Although the *linking* strategy might be effective in multitarget drug discovery, in principle, it generates high MW compounds, which might suffer of pharmacokinetic liabilities. Accordingly, the aforementioned *fusing* and *merging* strategies, leading to smaller hybrid molecules, are foreseen as more suitable to prevent such issues. 

In light of these considerations, we recently exploited a *fusing* approach to design a new series of hybrid compounds that combine the structural features of the ChEI donepezil with the metal chelator clioquinol (CLQ) [36]. 

We envisaged to integrate in the same molecule the anti-ChE properties with the neuroprotective effect provided by metal-driven oxidative stress inhibition, through chelation of redox-active metals. In fact, elevated concentrations of Cu(II) and Zn(II) have been detected in the neocortex of AD patients and are especially associated with β-amyloid (Aβ) deposits [37,38]. Binding sites for both metal ions have been identified on Aβ oligomers, and they are thought to mediate amyloid toxicity [39,40]. In fact, complexes of Aβ and metal ions were shown to promote Aβ aggregation [41] and protease resistance and to trigger the production of reactive oxygen species (ROS) [42]. On the basis of these observations, metal chelating therapy is considered an attractive option to counteract AD progression.

In this context, the 8-hydroxyquinoline (8HQ) derivatives CLQ and PBT2 were investigated for their neuroprotective effect in several neurodegenerative diseases [43,44,45,46]. Importantly, both CLQ and PBT2 showed promising therapeutic features in AD as metal-protein attenuation compounds (MPACs), capable of sequestering Cu(II) and Zn(II) from both amyloid plaques and the synaptic cleft, and operate as Cu(II) ionophores to balance the AD-related Cu(II) dyshomeostasis [47].

Interestingly, several other 8HQ-related compounds also showed neuroprotective activity [48], and the 8HQ scaffold was successfully exploited in multitarget programs for AD. In this respect, Rodriguez-Franco and co-workers reported on a series of tacrine-8HQ hybrids as novel MTDLs with cholinergic, antioxidant and Cu(II)-complexing properties [49]. Youdim *et al.* developed the hybrid compound M30, containing the metal chelator 8HQ core and the propargylamine moiety from FDA-approved anti-Parkinson rasagiline, with anti-MAO-B activity [50].

**Figure 3:**
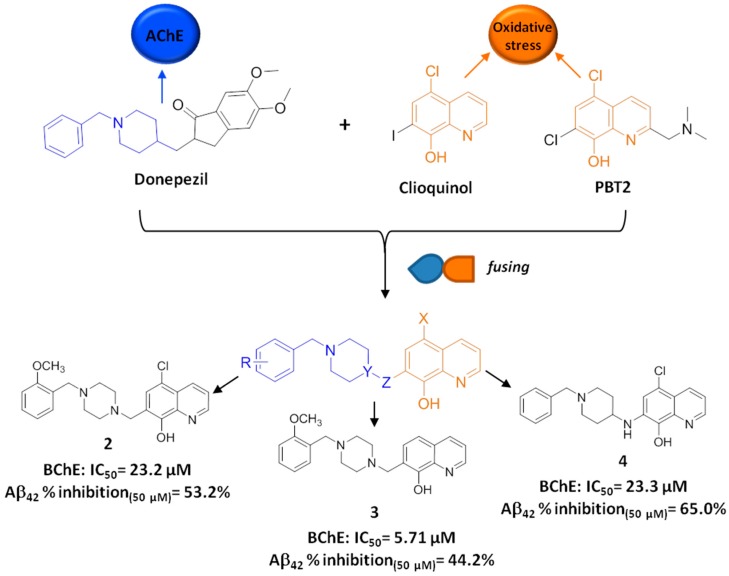
Design strategy (*fusing* approach) and multitarget activity of **2** to **4**.

Motivated by these considerations, some of us developed a novel series of hybrid compounds, rationally designed by fusing the 5-chloro-8HQ and the 8HQ nuclei with different benzylpiperidine-like moieties inspired by the AD drug donepezil [36] (Figure 3). We envisaged that by substituting the indanone core of donepezil with the planar and aromatic 8HQ one, we might have retained the affinity for the AChE PAS, as well as widened the spectrum of biological activities, together with a limited MW increase.

Thus, the new hybrid compounds were initially conceived of to exert a carefully-selected anti-AD MTDL profile: (i) ChE inhibition; (ii) Cu(II) and Zn(II) chelation; (iii) ROS scavenging; and (iv) Aβ anti-aggregating activity.

When evaluated *in vitro*, some of the synthesized compounds displayed a biological profile in compliance with the underpinning rationale. In particular, the majority of the new hybrids selectively targeted human butyrylcholinesterase (*h*BChE) at micromolar concentrations (5.71 ≤ IC_50_ ≤ 47.2) and effectively inhibited Aβ self-aggregation (% of inhibition at 50 µM ranging from 19% to 65%). In addition, compounds 5-chloro-7-((4-(2-methoxybenzyl)piperazin-1-yl)methyl)-8-hydroxyquinoline (**2**), 7-((4-(2-methoxybenzyl)piperazin-1-yl)methyl)-8-hydroxyquinoline (**3**) and 7-(((1-benzylpiperidin-4-yl)amino)methyl)-5-chloro-8-hydroxyquinoline (**4**) (Figure 3), with well-balanced anti-ChE and anti-aggregating activities, were also able to chelate Cu(II) and Zn(II) and exert antioxidant activity *in vitro*. Importantly, in the case of **3**, the multitarget profile was accompanied by positive blood-brain barrier (BBB) permeation in the parallel artificial membrane permeability assay (PAMPA), low cytotoxicity in T67 cells and acceptable toxicity in HUVEC primary cells.

Of note, compound **3**, fulfilling in a single chemical entity *in vitro* cholinergic, anti-aggregating, Cu(II)- and Zn(II)-chelating and antioxidant activities, might be worthy of additional investigation.

## 3. FBDD Applied to the Discovery of Anti-AD Lead Candidates

Fragment-based strategies have emerged as highly suitable for multitarget drug discovery [14]. Small fragments are particularly favourable starting points, since they might bind to multiple biological targets due to their inner chemical simplicity and be grown into lead-like molecules by step-wise addition of functional groups [9].

On these premises, we recently reported on a fragment-based program, which led to 6-amino-4-phenyl-3,4-dihydro-1,3,5-triazin-2(1*H*)-ones as the first class of dual-targeted compounds able to simultaneously modulate BACE-1 and glycogen synthase kinase-3 (GSK-3β) enzymes [51].

**Figure 4:**
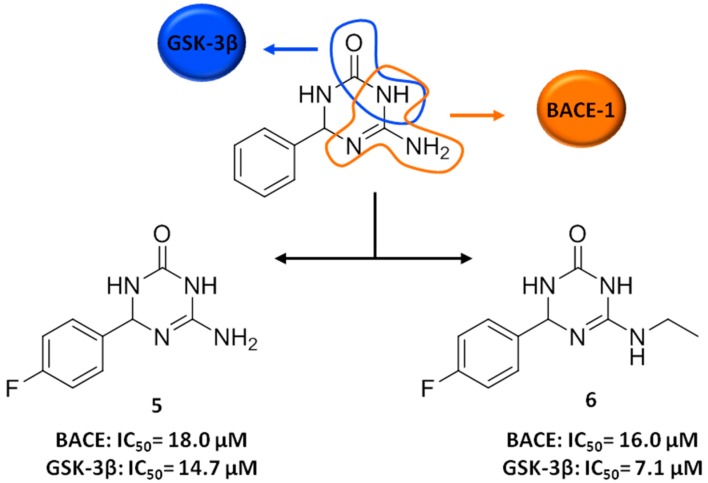
Design strategy (FBDD approach) and multitarget activity of **5** and **6**.

Particularly, we used a ligand-based approach to merge in a single pharmacophoric unit a guanidino motif and a cyclic amide group, as structural elements responsible for targeting BACE-1 and GSK-3β, respectively [51] (Figure 4). 

The guanidino moiety, a key functionality of several BACE-1 inhibitors, such as acylguanidines and aminoimidazoles, may bind to the catalytic aspartic dyad of BACE-1 [52,53]. Whereas, the amino and carbonyl functionalities of the cyclic amide group may act as H-bond donor and acceptor, respectively, thus forming H-bond interactions with the backbone of the GSK-3β hinge region. This cyclic amide function, present in numerous ATP-competitive inhibitors of GSK-3β, that is indirubins, maleimides and paullones, among others, is a signature for kinase binding, providing a specific H-bond network [54].

Among several others possible identified chemotypes, the 6-amino-4-phenyl triazinone core was selected as a suitable scaffold by means of molecular modelling studies [51].

On this basis, a preliminary structure-activity relationship (SAR) exploration of the triazinone core led to the fragment hit **5** (Figure 4) [51]. **5** showed balanced *in vitro* activities against both BACE-1 and GSK-3β enzymes (IC_50_ of 18.0 and 14.7 µM against BACE-1 and GSK-3β, respectively) [51]. This activity profile, although in the double-digit micromolar range, was considered particularly promising for several reasons. First, **5** displayed good LE metrics against both targets, as well as wide possibilities for further chemical tractability and functionalization. Second, in a network perspective, when connections exist between different targets, as appears to be the case for BACE-1 and GSK-3β, inhibitors with only moderate activities are predicted to have superior *in vivo* efficacy and fewer side effects than higher-affinity single-targeted compounds [16,50]. Moreover, a mild modulation of BACE-1 and GSK-3β activities has been reported to be sufficient to produce the desired therapeutic effect.

Importantly, **5** showed an interesting cellular profile in terms of neuroprotection, immunomodulation and neurogenesis [51]. In particular, **5** decreased nitrite production and neurotoxic activation in inflammation-insulted primary rat glia cells, resulting an interesting anti-inflammatory/immunomodulatory and neuroprotective agent. In fact, **5** was able to shift microglia phenotype from M1 to M2, with no changes in microglial phagocytic activity. This lends support to the innovative concept of targeting microglial cells by modulating their activity, rather than simply trying to counteract their inflammatory neurotoxicity [55]. The advantage of immunomodulation is that it reduces neuroinflammation and toxicity, while at the same time strengthening intrinsic neuroprotective properties of microglia and promoting neuroregeneration. Indeed, **5** was shown to differentiate neurosphere cultures of primary rat neural stem cells toward a mature neuronal phenotype. **5** also permeated the brain in mouse pharmacokinetic assessment, a fundamental property for central nervous system (CNS)-directed drugs [51].

Motivated by these interesting findings, the triazinone scaffold was further chemically manipulated providing the 6-(ethylamino)-4-(4-fluorophenyl)-3,4-dihydro-1,3,5-triazin-2(1*H*)-one **6**, as one of the most promising compounds of the whole series (Figure 4) [56]. Notably, **6**, with well-balanced potencies against the two isolated targeted enzymes (IC_50_ of 16.0 and 7.1 μM against BACE-1 and GSK-3β, respectively) displayed even better neuroprotective and neurogenic cellular activities than **5** and no neurotoxicity in cell-based assays. It also showed good brain permeability in a pharmacokinetic assessment in mice. Indeed, as, emphasized above, combining a well-balanced biological profile with drug-like pharmacokinetic properties is a critical challenge for multitarget drug discovery that has been met here [16].

## 4. Conclusions

All of the evidence we have accumulated so far corroborates the initial conviction that modulating a multiplicity of interconnected targets with an MTDL is an asset in treating a complex disorder of the elderly, such as AD. How should we move a concept that has been proposed and actively exploited for more than 10 years forward? Clearly, the aim is to develop a multitarget drug that could be brought to AD clinical trials. The case studies discussed above suggest that this is going to be a quite remote goal. With this background, the following should be among our priorities for the near future.

1. From an academic medicinal chemistry perspective, we need a focused effort to promote a deeper understanding of the mechanism of multitarget action and the use of a rigorous proof of concept. Robust pharmacokinetic/pharmacodynamic data should guide the on/off decision to further develop a hit candidate that truly possesses a multitarget profile, *i.e.*, (i) effectively reaches the brain; (ii) simultaneously modulates multiple targets and (iii) is more effective and safer than a single-targeted reference drug.
2. In terms of target selection, we need to move from targets, such as AChE and amyloid, which have been over searched in favour of other ones with more credential for a disease-modifying effect [57]. As a recent Nature article bluntly put it, “we are over-reliant on amyloid” and “the time has come to face our fears and reject the amyloid cascade hypothesis” [58]. Indeed, potentially more promising targets, such as tau, have remained largely under-explored so far.
3. Even more important is that the combination of targets to be addressed is made by using integrated network modelling and systems biology strategies. Multitarget approaches based on molecular network analysis may provide a fine-tuned modulation of the selected pathways instead of their complete blockade [51]. This appears to be critical for an effective therapeutic outcome.
4. Generally, turning a small molecule hit into a lead is a formidable challenge, as numerous hurdles beyond activity have to be overcome. This is even truer when dealing with multitarget drug discovery, given the added complexity of optimizing against multiple targets. Thus, it is vital to identify a high-quality starting hit for lead optimization, as this is crucial in determining the later potential for success [17].
5. Separate, although clearly related to this, is to push forward the use of cellular models as early as possible in the discovery phase. Different from isolated proteins, cell-screening systems maintain a reasonable experimental efficiency while preserving molecular-pathway interactions. Undoubtedly, this is indispensable in a network perspective. In particular, derivation of specific neural cells from patients’ induced pluripotent stem cells has made it possible to create *in vitro* models that recapitulate AD disease phenotypes. From a translation perspective, they represent unique platforms for AD (multitarget) drug discovery [59]. In this respect, treatment with approved or already validated combinations may provide clues or surrogate endpoints to track *in vitro*.

These are four ambitious objectives. Given the global increase of AD population and the frequent failures of drugs in clinical trials [60], attaining this goal is not only a formidable scientific challenge, but also an extremely important societal responsibility.

## Abbreviations

The following abbreviations are used in this manuscript:

8HQ	8-hydroxyquinoline
AChE	acetylcholinesterase
AChEI	acetylcholinesterase inhibitor
AD	Alzheimer’s disease
Aβ	β-amyloid
BACE-1	beta-secretasie-1
BBB	blood-brain barrier
CAS	catalytic anionic site
ChE	cholinesterase
CLQ	clioquinol
CNS	central nervous system
FBDD	fragment-based drug discovery
GSK-3β	glycogen synthase kinase-3
*h*BChE	human butyrylcholinesterase
HTS	high throughput screening
JNK3	c-Jun *N*-terminal kinase 3
LE	ligand efficiency
MAO	monoamine oxidase
MPACs	metal-protein attenuation compounds
MTDL	multitarget-directed ligand
MW	molecular weight
NMDAR	*N*-methyl-d-aspartate receptor
PAMPA	parallel artificial membrane permeability assay
PAS	peripheral anionic site
PPAR	peroxisome proliferator-activated receptor
ROS	reactive oxygen species
SAR	structure-activity relationships
